# Effect of Matrix-Modulating Enzymes on the Cellular Uptake of Magnetic Nanoparticles and on Magnetic Hyperthermia Treatment of Pancreatic Cancer Models In Vivo

**DOI:** 10.3390/nano11020438

**Published:** 2021-02-09

**Authors:** Felista L. Tansi, Filipp Fröbel, Wisdom O. Maduabuchi, Frank Steiniger, Martin Westermann, Rainer Quaas, Ulf K. Teichgräber, Ingrid Hilger

**Affiliations:** 1 Department of Experimental Radiology, Institute of Diagnostic and Interventional Radiology, Jena University Hospital—Friedrich Schiller University Jena, Am Klinikum 1, 07747 Jena, Germany; filipp.froebel@yahoo.de (F.F.); wisdom.maduabuchi@med.uni-jena.de (W.O.M.); 2 Center for Electron Microscopy, Jena University Hospital—Friedrich Schiller University Jena, Ziegelmuehlenweg 1, 07743 Jena, Germany; frank.steiniger@med.uni-jena.de (F.S.); martin.westermann@med.uni-jena.de (M.W.); 3 Chemicell GmbH, 12103 Berlin, Germany; quaas@chemicell.de; 4 Institute of Diagnostic and Interventional Radiology, Jena University Hospital—Friedrich Schiller University Jena, Am Klinikum 1, 07747 Jena, Germany; ulf.teichgraeber@med.uni-jena.de

**Keywords:** magnetic hyperthermia, pancreatic cancer, tumor microenvironment, hyaluronic acid, hyaluronidase, collagenase, magnetic nanoparticles

## Abstract

Magnetic hyperthermia can cause localized thermal eradication of several solid cancers. However, a localized and homogenous deposition of high concentrations of magnetic nanomaterials into the tumor stroma and tumor cells is mostly required. Poorly responsive cancers such as the pancreatic adenocarcinomas are hallmarked by a rigid stroma and poor perfusion to therapeutics and nanomaterials. Hence, approaches that enhance the infiltration of magnetic nanofluids into the tumor stroma convey potentials to improve thermal tumor therapy. We studied the influence of the matrix-modulating enzymes hyaluronidase and collagenase on the uptake of magnetic nanoparticles by pancreatic cancer cells and 3D spheroids thereof, and the overall impact on magnetic heating and cell death. Furthermore, we validated the effect of hyaluronidase on magnetic hyperthermia treatment of heterotopic pancreatic cancer models in mice. Treatment of cultured cells with the enzymes caused higher uptake of magnetic nanoparticles (MNP) as compared to nontreated cells. For example, hyaluronidase caused a 28% increase in iron deposits per cell. Consequently, the thermal doses (cumulative equivalent minutes at 43 °C, CEM43) increased by 15–23% as compared to heat dose achieved for cells treated with magnetic hyperthermia without using enzymes. Likewise, heat-induced cell death increased. In in vivo studies, hyaluronidase-enhanced infiltration and distribution of the nanoparticles in the tumors resulted in moderate heating levels (CEM43 of 128 min as compared to 479 min) and a slower, but persistent decrease in tumor volumes over time after treatment, as compared to comparable treatment without hyaluronidase. The results indicate that hyaluronidase, in particular, improves the infiltration of magnetic nanoparticles into pancreatic cancer models, impacts their thermal treatment and cell depletion, and hence, will contribute immensely in the fight against pancreatic and many other adenocarcinomas.

## 1. Introduction

A majority of solid cancers are characterized by aberrant and immunosuppressive tumor microenvironments (TME) with a highly dense extracellular matrix (ECM) [1,2,3]. The dense tumor ECMs are mainly composed of polysaccharides, fibrous proteins, glycoproteins, and proteoglycans, which include collagens, elastin, fibronectins, and hyaluronic acids (HA) amongst others [3,4,5]. Hyaluronic acids play a critical role in cancer relapse, which could be mediated, e.g., through their interaction with CD44, and the consequent activation of different cancer stem cell markers, multidrug resistance protein expression, micro-RNA expression and cancer stem cells renewal, survival, and drug resistance of different tumors [6,7]. Collagens, which are the major components of the ECM of most cancers and hence are key mediators of tumor desmoplasia, play key roles in tumor growth and therapeutic response. Different tumor types express variable numbers of the known 28 types of collagens, which depending on their interactions with membrane receptors such as integrins, glycoprotein VI, and receptor tyrosine kinases (e.g., discoidin domain receptors 1 and 2, DDR1/DDR2) induce signaling cascades that enhance tumor progression, metastasis formation, and chemoresistance (reviewed in [8]). This is, in part, due to a high interstitial pressure of desmoplastic tumors, which collapse blood vessels and hinder tumor accessibility to therapeutics [9]. The desmoplastic ECM is thus a key cause of poor treatment efficacy and tumor relapse. This is especially peculiar in cancers such as the pancreatic ductal adenocarcinomas (PDACs), which are hallmarked by dense fibrotic stroma, low blood vascularity and poor vascular perfusion [10] and are the second most difficult carcinomas to treat. Moreover, it could be shown that the degradation of collagen by matrix metalloproteinases provides proline-rich nutrients for the survival of PDACs under conditions of nutrient shortage [11]. Hence, a myriad of strategies have been developed to harness the TME and improve the tumor permeation and efficacy of different tumor therapies [2,12,13,14].

Although the implementation of a combination of different treatment modalities has led to an increase in the overall 5-year survival of PDAC from an initial < 5% to 8% in the past couple of years [15,16], PDAC recently became the second leading cause of cancer related mortalities in the United States, e.g., [16]. Leading factors for this include the late diagnosis and resulting nonresectability at the time of diagnosis, poor perfusion and low accessibility of the tumors to drugs [10] owing to tumor desmoplasia, and hence, poor response to therapeutic drugs [16,17]. Thus, the state of the art therapeutic modalities available are still insufficient for a better therapeutic success for PDAC. Considering this, magnetic hyperthermia has been studied extensively in preclinical models [18,19,20] and exposed promising potential as a new treatment technique for PDACs. Magnetic fluid hyperthermia (MH) has the potential to selectively deplete tumor cells in a minimal invasive manner and hence has gained entrance into several clinical trials [21,22,23]. MH exploits the ability of magnetic nanoparticles (MNP) to generate heat when placed in an alternating magnetic field, and consequently presupposes several basic requirements. Besides improving the specific heating potential of the nanoparticles used, e.g., through production as nanoclusters [24], the concentration of the magnetic nanoparticles at the target site is vital for an effective heat generation and thermal therapy for a given magnetic nanoparticle formulation and magnetic field feature [20,25]. Enhancing the uptake of magnetic nanoparticles into tumor cells or their perfusion into the tumor stroma would improve magnetic heating and subsequent thermal tumor therapy and tumor cell depletion. This is of therapeutic benefit for poorly treatable cancers such as pancreatic ductal adenocarcinomas [18] and breast carcinomas [19] amongst others. Nevertheless, the optimization of the MNP uptake into tumor cells or their penetration in dense tumor stroma is still a limiting factor that needs to be addressed. Different strategies to improve magnetic fluid accumulation into target sites include their functionalization with targeting ligands or therapeutic drugs for both intravenous and intratumoral application [19,26]. PDACs have high levels of collagens and hyaluronic acids [27,28,29,30], which contribute to their high rigidities and poor perfusion to drugs. Consequently, hyaluronidases [27], and collagenases [11] have been implemented in improving drug permeability in PDACs. Although a high level of hyaluronic acid and collagens are produced by stromal cells such as macrophages and fibroblasts, PDAC tumor cells also have high levels of intrinsic hyaluronic acid (HA) and collagen production [31,32], which, in part, is secreted to the extracellular matrix and stimulates cell motility. It can, therefore, be postulated, that implementing matrix-degrading enzymes like hyaluronidase and collagenase would enhance the deep penetration of tumors by magnetic nanoparticles, increase their uptake by tumor cells, and also improve the overall magnetic heating and tumor cell death upon exposure to an alternating magnetic field. 

In the underlying research work, we show that pretreatment of pancreatic cancer cell lines and 3D cultures thereof with hyaluronidases and collagenase improved the overall uptake of MNP into the cells and their subsequent hyperthermia treatment. Furthermore, in in vivo mice models of pancreatic cancer xenografts, hyaluronidase influenced the relaxation of the tumor stroma and the infiltration and distribution of a cell-impermeable large dextran-coated magnetic nanoparticle formulation (Dex-MNP) as a consequence. The results expose that the consequence of an improved hyaluronidase-based Dex-MNP infiltration of the tumor stroma is a lower overall magnetic heat dose (calculated as the cumulative equivalent minutes of the tumors at 43 °C, CEM43) as compared to hyperthermia treatment without hyaluronidase usage, which exposes a more homogeneous distribution of heat due to Dex-MNP distribution. Although several reports expose that different cancer cells, including breast [33] and pancreatic cancer [18] cells, show resistance to low heat doses, the use of hyaluronidase showed a gradual, but persistent tumor growth inhibition despite the comparably lower heat dose than achieved with MH without enzymes. Taken together, the results reveal potential benefits of implementing hyaluronidases for the improved infiltration of magnetic nanofluids into the tumor stroma and cells for magnetic hyperthermia purposes. This will reduce overheating discomfort for patients and also circumvent the formation of heat spots due to inhomogeneous distribution of the nanofluids within tumors and the consequent regrowth of insufficiently heated tumor cell niches. Furthermore, it would enhance tumor growth depletion due to the effect of hyaluronidase on the inhibition of hyaluronic acid-based signaling, chemoresistance, and tumor relapse. The implementation of the hyaluronidase-based nanofluid infiltration and magnetic hyperthermia has the potential to simultaneously improve the efficacy of other therapeutic drugs when used in combination.

## 2. Materials and Methods 

### 2.1. Cell Culture and Preparation of 3D Spheroids

Pancreatic ductal adenocarcinoma cell lines (Panc-1 and BxPC-3) were cultured at standard culture conditions (37 °C, 5% CO_2_ and 95% humidity) in DMEM and RPMI media supplemented with 10% fetal calf serum (FCS), respectively. Media and FCS were from Invitrogen GmbH (Karlsruhe, Germany). Except otherwise indicated, 3D cancer spheroids were prepared in large numbers in 500 mL in 3D culture spinner flasks (Pfeiffer Electronic Engineering GmbH, Germany) or in smaller amounts in 96-well tissue culture plates (precoated with 50 μL of 2% low melting point agarose in culture medium, to prevent adherence of the cells). Briefly, cells grown as monolayer cultures were dissociated using trypsin/EDTA then counted. At least 1 × 10^7^ cells were transferred to a spinner flask containing 250 mL prewarmed culture medium. The spinner flasks were placed on a magnetic stirrer (Pfeiffer Electronic Engineering GmbH, Germany) in a cell culture incubator and the cells were cultured for 4–10 days at standard culture conditions under constant stirring at 40 revolutions per minute. Medium change was performed by replacing 100 mL medium with fresh one every 2 days, after sedimentation of the spheroids. To culture spheroids in 96 well tissue culture plates, 5 × 10^3^ cells/well were seeded in 200 µL medium per well of the precoated 96-well plates, then centrifuged (200× *g*, 5 min, 25 °C) to initiate the formation of the spheroids and cultured for 4–10 days at standard culture conditions with replacement of 100 µL medium/well with fresh medium every 2 days. The spheroid morphology and sizes were controlled every 2 days by light microscopy.

### 2.2. Characterization of Nanoparticle Sizes and Morphology

Two different MNP formulations were implemented. Starch-coated iron oxide nanoparticles (fluidMAG/C11-D from Chemicell GmbH, Berlin, Germany) and Dextran-coated iron oxide nanoparticles (RCL01, (termed Dex-MNP herewith) from Resonant Circuits Limited, London, UK) were used for the in vitro cellular and in vivo tumor studies, respectively. The hydrodynamic diameter (Z-averages), zeta potentials, and polydispersity indices were determined by dynamic light scattering (DLS) on a Zetasizer Nano ZS (Malvern Instruments, Herrenberg, Germany), whereas the morphology and core sizes of the nanoparticles were validated by transmission electron microscopy (TEM; Philips CM 120, Eindhoven, The Netherlands). 

### 2.3. Determination of the Specific Absorption Rate (SAR) and Intrinsic Loss Power of MNP 

The SAR values of MNP dispersed in 200 µL water at a concentration of 4 mg Fe/mL were deduced by exposure to an alternating magnetic field, AMF (*H* = 8.49 ± 0.10 kA/m, *f* = 1.048 ± 0.01 MHz) and measuring the initial temperature rise with a fiber-optic temperature sensor system (TS5 and FOTEMPMK-19, Optocon AG, Germany). The SAR values were calculated using the formula:

(1) $$SAR=C*\frac{MF}{mp}*\frac{\Delta T}{\Delta t},$$

where *C* is the specific heat capacity of the MNP dispersion, m_F_ and m_p_ represent the masses of the fluid and nanoparticle iron, respectively, and ΔT/Δt the maximum value of the linear slope monitored immediately after switching on the alternating magnetic field. The intrinsic loss power (ILP) was calculated by using the formula:

(2) $$ILP=\frac{SAR}{\left(H^{2}\;x\;f\right)},$$

where *H* is the magnetic field strength and *f* is the frequency of the system used. In this case, the MACH-System Model2017Jena (Resonant Circuits Limited, RCL London, UK) was used at a field amplitude *H* = 8.49 ± 0.10 kA/m and frequency *f* = 1.048 ± 0.01 MHz. 

### 2.4. Quantification of the Whole Iron Content in Nanoparticles and Pancreatic Cancer Cells

The iron content of MNPs and also cells and 3D spheroids after exposure to MNP was quantified by flame atomic absorption spectroscopy (AAS 5 FL; Analytik Jena AG, Jena-Germany) as described in more details in the supplementary methods in [34]. For the determination of iron levels in MNP fluids, 50 µL of the MNP-suspension was placed in a 2 mL reagent tube and then homogenized on a vortexer after adding 1 mL of a 32% hydrochloric acid, HCl (Roth). The samples were incubated for 30 min at room temperature, diluted at 1:441 with distilled water, and subjected to protein precipitation. For this, 1 part of a 32% HCl solution was added to 2 parts of the homogenized, /diluted MNP sample and then one part of a 10% TCA solution was added. The mixture was homogenized by vortexing, and then, it was centrifuged for 5 min at 2348× *g*. The supernatants were used for AAS measurements, conducted in triplicate. 

Flame atomic absorption spectroscopy (FAAS), determines whole iron (II/III) oxide, hence, this was considered in determination of iron in cells and 3D spheroids. For this, control cells and 3D spheroids not exposed to MNPs were used for subsequent normalization to get the iron levels originating from the MNP. Equal number of cells (10–20 × 10^6^ cells) for both the hyperthermia-treated and control samples were used. These were pelleted in 2 mL reagent tubes and were then lysed by adding 500 µL of a 32% HCl solution, and then, they were subjected to shaking at 1000 rpm for 30 min at 75 °C. Subsequently, 500 µL of a 10% TCA solution was added for protein precipitation, and the tubes were further incubated for 15 min. The resulting solutions were diluted at 882-fold with distilled water and measured spectroscopically in triplicate (AAS 5 FL, Analytik Jena). The total iron content of the samples was determined based on a calibration standard with known iron concentrations (0, 5, 10, 20, 30, and 50 µmol Fe/L in 37% HCl) using the formula:

(3) $$\frac{\mathrm{Mg}}{g}Fe\;\left(sample\right)=\frac{Fe\left[\frac{mmol}{L}\right]xdilution\;factor\;x\;molar\;mass\;Fe\left[g/mol\right]}{X}$$

where *Fe* [*mmol/L*] is the result of FAAS measurement and *X* is the number of cells used or concentration of MNP ferrofluid (in mL) used. 

### 2.5. Treatment of Cells and 3D-Spheroids with Matrix-Degrading Enzymes and MNP

Except otherwise indicated, Panc-1 or BxPC-3 cells grown to 80% confluency on 8-well culture slides or tissue culture flasks (BD Biosciences, Bedford MA, USA), and 3D spheroids (Panc-1, 79 single spheroids equivalent to 2 × 10^6^ cells) cultivated for 7 days in 3D culture spinner flasks were washed once with HBSS (Biochrom GmbH, Berlin, Germany) and 3 times with serum-free culture medium. The cells were supplemented with serum-free culture media containing 40 µg/mL bovine testes hyaluronidase I-S or hyaluronidase IV-S (Sigma Aldrich, Missouri USA) or 20 µg/mL to 40 µg/mL collagenase, (clostridopeptidase A, from Sigma Aldrich) and further cultured for 4 h at standard culture conditions. Thereafter, the medium was removed and complete medium supplemented with MNP at different concentrations ranging from 5 to 50 µg Fe/mL were added and the cells further cultured for 2–24 h. Cells aimed for subsequent magnetic hyperthermia treatment were grown in large tissue culture flasks, treated with enzymes and exposed to MNP (50 µgFe/mL) as described above. Thereafter, the cells were washed 3 times with HBSS to removed free MNPs, and dissociated with Biotase (Biochrom GmbH Berlin, Germany), and counted, and 1 × 10^7^ cells were pelleted and dispensed in 200 µL prewarmed complete culture medium for exposure in an alternating magnetic field.

### 2.6. Qualitative Analysis of MNP Localization in Cells and 3D Spheroids by Prussian Blue Staining

In order to qualitatively evaluate the levels of MNP uptake in cells by microscopy, 20,000 cells (BxPC-3) or 30,000 cells (Panc-1) were seeded on 8-well culture chamber slides, subsequently cultured, and incubated with enzymes and MNPs as indicated above. Thereafter, the cells were harvested by washing 3 times with HBSS to remove noninternalized MNP. The cells were fixed for 30 min at room temperature (RT) with 3.7% formaldehyde in HBSS, then washed and stained by the Prussian blue method. Furthermore, 3D spheroids were embedded in 1% agarose and then paraffinized, and 5–10-µm thick sections were sliced and dewaxed 2 times by 10 min incubation in toluene followed by fixation /rehydration in a series of 90%, 70%, and 50% ethanol. Subsequently, the dewaxed 3D spheroid slices and fixed monolayer cells were incubated for 10 min in 10% potassium ferrocyanide and then for 30 min in a mixture of 20% hydrochloric acid and 10% potassium ferrocyanide (both from Sigma Aldrich, Steinheim Germany). Finally, the cytoplasma were counter-stained with Eosin B solution. All slides were mounted with Faramount (Dako, Glostrup, Germany) and cover-slipped, and the cells were imaged on an Olympus BX50 microscope.

### 2.7. Magnetic Hyperthermia Treatment of Cultured Cells

Panc-1 cells grown in tissue culture flasks and exposed to matrix-degrading enzymes and MNP and pelleted as described above were exposed to an AMF (*H* = 8.49 ± 0.10 kA/m, *f* = 1.048 ± 0.01 MHz) in order to induce temperatures of 43 °C for 60 min. Temperatures were monitored with a fiber-optic temperature sensor system (TS5 and FOTEMPMK-19, Optocon AG, Germany).

### 2.8. Animal Studies

The use of animals was approved by the regional ethical committee for animal control and care (Thüringer Landesamt für Verbraucherschutz, Bad Langensalza, Germany under the number UKJ-17-030 of 12 October 2017) and conformed to the international guidelines on the ethical use of animals. For tumor induction, approximately, 8–10-weeks old female nude mice (Rj:Athym-Foxn1nu/nu, Janvier, Germany) were injected subcutaneously on the lower back with 2 × 10^6^ cells of the Panc-1 pancreatic cancer cells, dispensed in 100 µL cold Matrigel^®^ (Corning, Kaiserslautern, Germany). Mice were subsequently maintained under standard conditions (14 h/10 light–dark cycles; 25 °C temperature) with ad libitum food and water supply. Mice were subjected to therapy when the tumors reached an average of 5–10 mm diameter. 

### 2.9. Application of Enzymes, Magnetic Nanoparticles, and Magnetic Hyperthermia Treatment of Tumor Models in Mice

Mice bearing subcutaneous Panc-1 tumors of 5–10 mm diameters were randomly divided in 3 groups of 5 mice/group and intratumorally injected with the large dextran-coated magnetic iron oxide nanoparticle (Dex-MNP), which, due to a large size (149 nm and a core size of approximately 50 nm, cannot be taken up by macrophages and cancer cells in vitro. Hence, their use in the in vivo situation will provide a better evaluation of the enzyme-induced infiltration of magnetic nanoparticles into the tumors. The mice groups were as follows: Group 1 mice (termed 24 h) received Dex-MNP alone and a waiting time of 24 h before exposure to the first of 2 alternating magnetic field exposure sessions. Group 2 mice (termed Hya/24 h) received Dex-MNP at 2 h post intratumoral injection of hyaluronidase-I-S (40 µg/100 mm^3^ tumor volume) and a waiting time of 24 h prior to the first hyperthermia treatment, whereas mice in group 3 (termed 72 h) were intratumorally injected with Dex-MNP alone and given a waiting time of 72 h before the first exposure to magnetic hyperthermia in order to grant longer time for the nanofluid infiltration. For each MNP injection, 0.5 mg Fe/100 mm^3^ tumor volume was applied. Magnetic hyperthermia treatment was performed by exposing tumors to an alternating magnetic field, AMF (H = 5.43 to 8.49 kA/m, f = 1.048 MHz) for 60 min. Thereby, 2 treatment sessions were implemented, the first being 24 h after MNP injection (Groups 1 and 2) or 72 h after injection (Group 3), and the second treatment followed 7 days after the first hyperthermia treatment. During hyperthermia treatments, the tumor and body temperatures were monitored simultaneously with a fiber-optic temperature sensor (TS5 and FOTEMPMK-19, Optocon AG, Dresden, Germany) and an infrared thermography camera (InfRec R300, Nippon Avionics Co., Yokohama, Japan). The acquired temperatures were used to derive the thermal doses (cumulative equivalent minutes, CEM43) applied to the tumors as described below. Treatment outcome was determined based on body weight and tumor volume (caliper) measurements for an overall 30 days from the day of the first hyperthermia treatment and on blood count validation on day 30 of therapy. 

### 2.10. Determination of the Magnetic Hyperthermia-Induced Thermal Dose (CEM43)

The thermal dose, deduced as the cumulative equivalent minutes above 43 °C (CEM43), was determined according to Sapareto and Dewey, using the formula:

(4) $$\mathrm{CEM}43=\overset{n}{\underset{i=1}{\sum }}t_{i}\times R^{\left(43-T_{i}\right)}$$

The CEM43 is the equivalent time that the cells must be treated at 43 °C in order to achieve the same thermal effect as with a varying temperature. *t_i_* is the time duration of measurement at a designated temperature *T_i_*. R is a constant that is equal to 0.5 when temperature T > 43 °C and equal to 0.25 at T < 43 °C. If T is < 37 °C, then R = 0 and the CEM43 also becomes zero. This ensures that the CEM43 is only calculated when temperatures higher than body temperatures are achieved. 

### 2.11. Computer Tomographic Detection of Magnetic Ferro Fluids in Mice 

Mice anesthetized with 2.5 Vol% isoflurane, were scanned on an IVIS^®^ Spectrum CT (Perkin Elmer, Waltham, MA, USA) using the medium resolution setup (225 µm resolution, 130 mGy X-ray dose). Tomographic images were acquired after injection of the Dex-MNP and at different time points during therapy by magnetic hyperthermia. The acquired datasets were evaluated with the Living Image^®^ software Version 4.3.1., (Perkin Elmer, Waltham, MA, USA). Thereby, 3D images were reconstructed using the 3D multimodality tool applying histogram adjustment based on the X-ray density of bone and the magnetic Dex-MNP. 

### 2.12. Blood Collection and Analysis 

Mice undergoing euthanasia under 5 Vol% isoflurane were carefully incised along the neck region and the salivary glands were removed. The subclavian vein was then punctured and blood was collected with a 50 µL Na-heparin capillary (Hirschmann GmbH & Co. KG, Eberstadt, Germany), and it was quickly transferred to a 200 µL isotonic salt solution (Fresenius Kabi GmbH, Bad Homburg, Germany) and measured on an automated hematology system for animals (Sysmex XT-1800i, Sysmex corporation, Kobe, Japan) according to the user manual. 

### 2.13. Ultrathin Section Transmission Electron Microscopy

After hyperthermia treatment and euthanasia, the residual tumor tissues were excised. Pieces of tissue of 1 mm size were fixed for 3 h at room temperature and then overnight at 4 °C with 4% (*v*/*v*) formaldehyde (freshly prepared from paraformaldehyde) and 2.5% (*v*/*v*) glutaraldehyde in sodium cacodylate buffer (0.1 M and pH 7.2). The tissue samples were washed 3 times for about 30 min with sodium cacodylate buffer and postfixed with 1% (*w*/*v*) osmium tetroxide in cacodylate buffer for 2 h at 20 °C. Samples were dehydrated in an ascending ethanol series and stained with 2% (*w*/*v*) uranyl acetate in 50% (*v*/*v*) ethanol. The samples were embedded in Araldite resin (Plano, Wetzlar, Germany) according to manufacturer’s instruction. Ultrathin sections of 70 nm thickness were cut using an ultramicrotome Ultracut S (Reichert-Jung, Vienna, Austria) and mounted on Formvar-carbon-coated 100 mesh grids (Quantifoil, Großlöbichau, Germany). The Ultrathin sections were stained with lead citrate for 10 min and examined in a Zeiss EM 902A electron microscope (Carl Zeiss AG, Oberkochen, Germany) operated at 80 kV. Digitized images were taken with a Wide-angle Dual Speed 2K CCD camera controlled by a Sharp:Eye base controller and operated by the Image SP software (camera and software: TRS, Moorenweis, Germany).

### 2.14. Cell Viability Assays

Cell viability was derived based on the ability to reduce the non-fluorescent resazurin to the red fluorescent resorufin post hyperthermia treatment. For this purpose, 6 × 10^3^ cells (control or magnetic hyperthermia-treated cells in 100 µL culture medium) were seeded per well into 96-well plates (Greiner Bio-One GmbH, Frickenhausen, Germany) in sextuple and cultured under standard conditions for 24 or 48 h. Thereafter, the cells were washed three times with HBSS, and then, 10 % (*v*/*v*) of alamarBlue^®^ cell viability reagent (Fischer scientific GmbH, Schwerte, Germany) in culture medium was added; further, the plates were incubated at standard conditions for 2 h. The fluorescence was measured at 590 nm, using the Infinite M1000 PRO plate reader (Tecan Austria GmbH, Grödig, Austria) after exciting at 530–560 nm. The deduced fluorescence of the cells after hyperthermia treatment was normalized to values obtained for control untreated cells. Except otherwise indicated, each experiment was performed three independent times.

### 2.15. Colony Formation Assay

After hyperthermia treatment, cells were dispensed in 6-well plates at a density of 5 × 10^3^ cells, and then, cultured at standard culture conditions for 2 weeks with regular medium change twice per week. All samples were prepared in duplicates. After two weeks, the cells were washed and stained with coomassie blue or crystal violet solution (Cell Biolabs Inc., Lorrach, Germany); the number of colonies that resulted from surviving cells were counted, and the relative area of the plate occupied by the colonies was deduced using the open-source GSA ImageAnalyser software.

### 2.16. Statistical Analysis

Data requiring statistical evaluations were analyzed in SigmaPlot 14.0 program using the student’s *t*-test to compare groups. In vitro data are presented as mean of 3 independent experiments ± standard deviations (S.D.), whereas in vivo data are presented as mean of n ≥ 4 mice/group and the standard error of means (SEM). Differences with *p* < 0.05 were considered statistically significant.

## 3. Results

### 3.1. Physical Parameters of the Iron Oxide Nanoparticles

**Figure 1:**
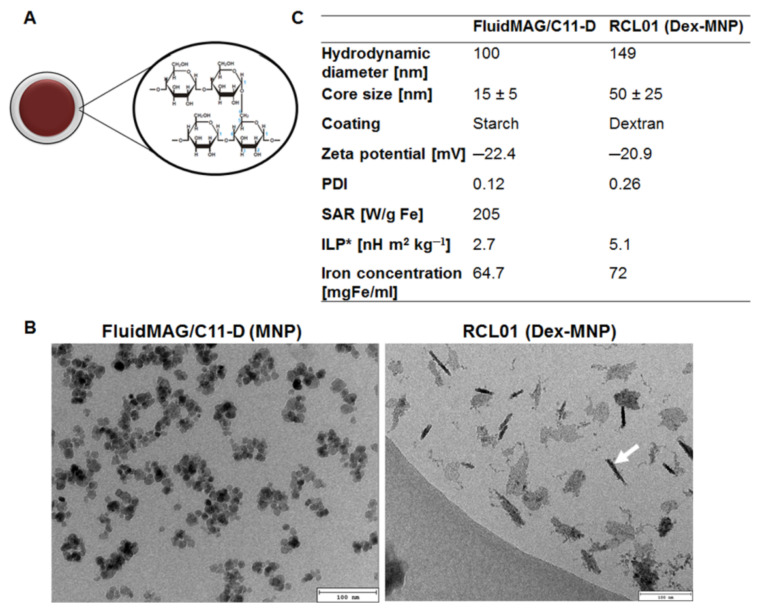
Characterization of magnetic nanoparticles. (**A**) Schematic presentation of the FluidMAG/C11-D particle core composed of Magnetite-C11 and starch coating. (**B**) Transmission electron micrographs of the nanoparticles revealing the particle core of approximately 15 and 50 nm diameter for the FluidMAG/C11-D and RCL01 (Dex-MNP), respectively. The white arrow points at a Dex-MNP lying perpendicular to the plane. Scale bars 100 nm. (**C**) Characteristic properties of the nanoparticles depicting hydrodynamic diameter, zeta potential, and polydispersity indices (PDI) deduced by dynamic light scattering; the iron concentration deduced by atomic absorption spectroscopy (AAS); and the specific absorption rate (SAR) and intrinsic loss power (ILP) determined with an AC frequency *f* = 1.048 MHz and field amplitude *H_o_* = 8.49 kA/m for the FluidMAG/C-11D.

Iron oxide-based magnetic nanoparticles (FluidMAG/C11-D) were coated with starch as depicted in the schematic model (Figure 1A). They conveyed average core sizes of 15 nm, whereas the dextran-coated iron oxide particles (Dex-MNP) showed flake-like cores of approximately 50 nm diameters (Figure 1B). The iron content of the nanoparticles was 64.7 mg Fe/mL and 72 mg Fe/mL fluid, whereas the hydrodynamic diameters were 100 nm and 149 nm, respectively. The specific absorption rate (SAR) of the starch-coated FluidMAG/C11-D particles determined on a low frequency (1.048 MHz) alternating magnetic field, and a field amplitude, *H* of 8.49 kA/m was 205 W/gFe, and the corresponding intrinsic loss power (ILP) was 2.7 nHm^2^/kg (Figure 1C). In comparison, the ILP of the RCL01 (Dex-MNP) as determined by the supplier was 5.1 nHm^2^/kg.

### 3.2. Hyaluronidases and Collagenase Enhance Uptake of MNP by Pancreatic Cancer Cells

**Figure 2:**
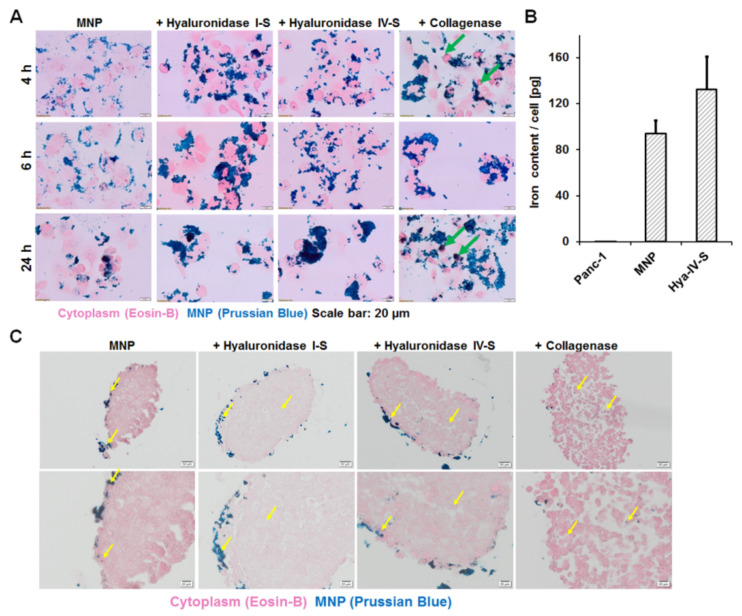
Matrix-modifying enzymes cause increased cellular uptake of MNP in pancreatic cancer cells. (**A**) Representative light microscopic images of Prussian blue-stained MNP in pancreatic cancer cells pretreated with 40 µg/mL of the indicated hyaluronidases or 20 µg/mL of collagenase in serum-free medium for 2 h, before incubation with 10 µg Fe/mL MNP in growth media containing FCS for the indicated duration. Collagenase caused a higher rounding of the cells from the culture vessels (green arrows) than the hyaluronidases. (**B**) Comparative levels of iron per cell after exposure to MNP without or with pretreatment with hyaluronidase IV-S (Hya-IV-S), n = 3/S.D., *p* = 0.09 for MNP versus Hya-IV-S. (**C**) Representative light microscopic images of Prussian blue-stained 3D spheroid slices showing characteristic blue stain of MNP-based iron without or with pretreatment with 40 µg/mL of hyaluronidases or 20 µg/mL of collagenase in serum-free medium for 4 h before exposure to 50 µg Fe/mL of the MNP in complete growth media for 24 h. MNP alone shows no loosening of the spheroid stroma and MNP only on the spheroid surface. The hyaluronidases caused moderately dissociated stroma and very high MNP uptake by cells lining the spheroid surface and a few in the spheroid center (yellow arrows), whereas collagenase caused strongly dissociated stroma and deep MNP-infiltration. Scale bars: upper images 50 µm and lower images 20 µm.

Pretreatment of pancreatic ductal adenocarcinoma cells (Panc-1) grown as monolayer culture, with the matrix-degrading enzymes hyaluronidase I-S, hyaluronidase IV-S, and collagenase prior to exposure to starch-coated MNP suspension improved the speed and level of uptake of the MNP. As seen from concentration-dependent MNP uptake studies (Supplementary data S1), the use of 10 µg Fe/mL of the MNP suspension revealed the most reliable levels of uptake after 24 h. Hence, the impact of the enzymes on MNP uptake by Panc-1 cells was accessed using 10 µg Fe/mL MNP suspension. A higher uptake of MNP after treatment with different enzymes was evident in the intensity of Prussian-blue-stained nanoparticulate iron within the cells (Figure 2A). Interestingly, collagenase-treated cells revealed the highest intensities of stained iron after 4 h incubation than the hyaluronidase I-S and hyaluronidase-IV-S-treated cells. Moreover, collagenase caused a greater level of rounding of the cells, which are indicative of proliferation or apoptosis (Figure 2A, green arrows). Quantification of the total iron content of cells pretreated with hyaluronidase IV-S before exposure to starch-coated MNP revealed an average 132 pgFe/cell as compared to 94 pgFe/cell for MNP alone (Figure 2B), although with only a tendency (*p* = 0.09) of statistical difference. Contrarily, nontreated Panc-1 cells showed below 1 pgFe/cell.

Although the hyaluronidases caused moderate loosening of the stroma of 3D spheroids and improved infiltration and uptake of MNP as compared to controls without enzyme, collagenase caused a strong dissociation of the cells from each other in 3D-spheroids (Figure 2C). Contrarily, MNP-treated Panc-1 cells showed uptake of MNP only on the spheroid surface.

### 3.3. Enzyme-Induced Increases in MNP Uptake Influences Magnetic Heating and Subsequent Death of Pancreatic Cancer Cells

**Figure 3:**
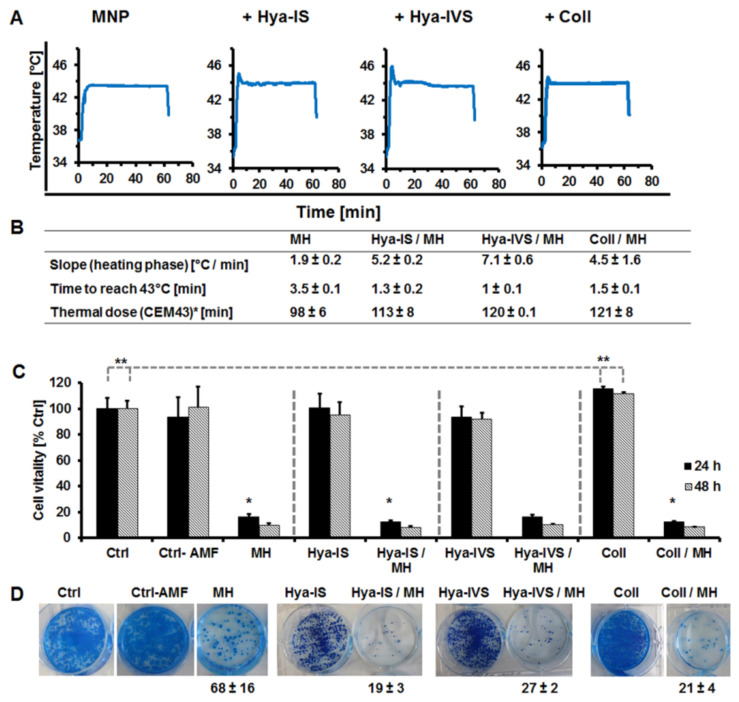
Influence of matrix-modulating enzymes on magnetic hyperthermia treatment of pancreatic cancer cells. (**A**) Representative heat curves of cells during exposure to an alternating magnetic field (AMF). (**B**) Cells treated with MNP alone (MH) reveal slower increase in temperature upon exposure to an AMF and lower overall heat dose (CEM43) after 60 min than those pretreated with hyaluronidase-IS (Hya-IS/MH), hyaluronidase-IVS (Hya-IVS/MH), or collagenase (Coll/MH) before incubation with MNP n = 3/S.D. (**C**) Relative vitality of cells grown for 24 and 48 h after magnetic hyperthermia treatment. * *p* < 0.05 signifies lower cell viability (higher cell death) for Hya-IS-/MH- and Coll-/MH-treated cells as compared to MH-treated cells. ** *p* ≤ 0.036 signifies higher cell viability (proliferation) for 24 and 48 h collagenase (coll)-treated cells as compared to control untreated Panc-1 cells. Each bar depicts the mean of n = 4/S.D. for control cells (Ctrl) and n = 3/S.D. for the treatment groups. (**D**) Representative pictures of well plates showing cells monitored for colony formation for 2 weeks after magnetic hyperthermia treatment. 5 × 10^3^ cells were seeded per well. Enzyme-/hyperthermia-treated cells show higher (*p* < 0.05) cell death and inability to form colonies than cells treated with MNP and hyperthermia (MH) alone.

The relatively increased level of MNP uptake by Panc-1 cells was indirectly seen in a rapid increase in temperatures of cultured cells after exposure to the same AC frequency and amplitude of an alternating magnetic field (Figure 3A). Accordingly, the temperature increase (slope) during the heating phase was 1.9 ± 0.2 °C/min for the cells exposed to MNP alone, whereas the enzyme-treated cells showed 5.2 ± 0.2, 7.1 ± 0.6, and 4.5 ± 1.5° C/min heating for the hyaluronidase-I-S, hyaluronidase-IV-S, and collagenase, respectively (Figure 3B). Starting from an initial average temperature of 36 °C, the time for the cells to reach 43 °C was significantly different (*p* < 0.001) for enzyme treated as compared to MNP alone. Hence, 3.5 ± 0.1 min was determined for cells exposed to MNP alone, but reduced to 1.3 ± 0.2, 1 ± 0.1, and 1.5 ± 0.1 min for the cells pretreated with hyaluronidase-I-S, hyaluronidase-IV-S, and collagenase, respectively, prior to exposure to MNP. Although the control MNP-treated cells were exposed to the maximum possible amplitude of the magnetic hyperthermia device used but did not exceed 43 °C heating, the rapid and high increases in temperatures of cells pretreated with enzymes and MNP required adaptation of the heating with lower amplitudes in order to maintain 43 °C temperatures for the overall duration of treatment. Thus, the calculated thermal dose after 60 min treatment of cells (given as cumulative equivalent minutes above 43 °C, CEM43) was 98 ± 6 min for the cells treated with MNP alone as compared to 113 ± 8, 120 ± 0.5, and 121 ± 8 min for the cells pretreated with hyaluronidase-I-S, hyaluronidase-IV-S, and collagenase, respectively. The enzyme treatment caused a significant increase in the thermal doses (*p* = 0.047 for Hya-IS, *p* = 0.003 for Hya-IVS, *p* = 0.014 for Coll) as compared to MNP alone. 

Also, a high thermal induction of cell death was observed in all MNP and hyperthermia-treated cell samples (Figure 3C). Interestingly, cells treated with hyaluronidase-I-S and collagenase before MNP exposure revealed significantly lower (*p* < 0.05) cell viabilities than the cells that were treated with MNP alone. Contrarily, hyaluronidases alone (Figure 3C, Hya-IS and Hya-IVS) had no influence on cell viability, whereas treatment with collagenase alone intriguingly induced proliferation of the cells significantly, (** *p* = 0.036 at 24 h and 0.031 at 48 h) as compared to the untreated Panc-1 cells (Figure 3C, Coll). However, less colonies were formed by cells treated with MNP and enzymes before hyperthermia than for those treated with MNP alone before magnetic hyperthermia. Accordingly, after culturing for 2 weeks post hyperthermia treatment, cells treated with hyaluronidases in combination with magnetic hyperthermia (Figure 3D, Hya-IS/MH and Hya-IVS/MH) or with collagenase and magnetic hyperthermia (Figure 3D, Coll/MH) lead to the formation of fewer colonies (*p* < 0.001), namely, 19 ± 3, 27 ± 3, and 21 ± 4 colonies, respectively, as compared to cells treated with MNP alone prior to hyperthermia, (Figure 3D, MH) which revealed an average survival and growth of 68 ± 16 visibly larger colonies. Taken together, the results show that the use of matrix-modulating enzymes increased MNP uptake, improved magnetic heating, and showed a higher level of cell death and a lower ability of the cells to regrow and form colonies post hyperthermia treatment. 

### 3.4. Effect of Matrix-Modulating Enzymes on the Infiltration of MNP and Magnetic Heating of Tumor Models in Mice

**Figure 4:**
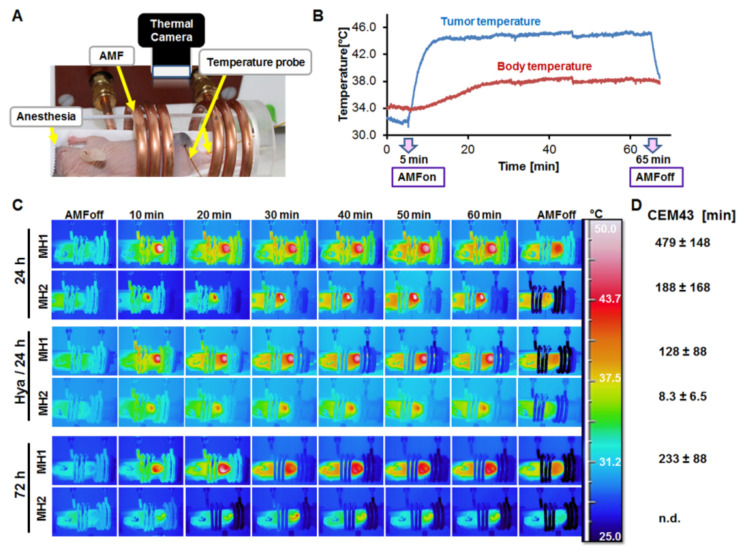
Influence of hyaluronidase on magnetic hyperthermia treatment of pancreatic cancer models in mice. All mice received comparable concentrations (0.5 mg Fe/100 mm^3^ tumor volume) of intratumoral Dex-MNP. (**A**) Setup of mice in the alternating magnetic field (AMF). (**B**) Representative temperature curve of tumor and mouse body showing increase and maintenance of heat above 43 °C in the tumor when the AMF is on and the decrease when the AMF is switched off. (**C**) Representative thermographic images of mice treated with the first magnetic hyperthermia (MH1) and the second hyperthermia (MH2) 7 days after. (**D**) Mean and standard deviations of the calculated heat doses (CEM43) per treatment group (n ≥ 3/S.D.).

Tumor-bearing mice were randomly grouped in 3 groups of 4–5 mice each and injected intratumorally with the dextran-coated MNP (Dex-MNP) formulation, which could not be taken up by cultured macrophages and pancreatic cancer cells (Supplementary data S2). To study the effects of enzymes, the Dex-MNP was applied 2 h after pre-injection of hyaluronidase-I-S (40 µg/100 mm^3^ tumor volume) as described in detail under the Material and Methods section. Two mice groups received only the Dex-MNP injection and differed in the duration of Dex-MNP infiltration time (24 versus 72 h) before the first exposure to an AMF. The group receiving hyaluronidase (Hya/24 h) was left for 24 h before the first AMF exposure. After the respective duration after Dex-MNP injections, the mice were placed in an AMF (Figure 4A) and thermally treated for 60 min after an adaptation time of 5 min. As can be seen in the heating curve of temperatures monitored with a thermal sensor probe, the temperatures of the tumors rise to 43–44 °C when the alternating magnetic field is switched on, and decrease rapidly when the AMF is switched off (Figure 4B), whereas the body temperatures are not greatly affected. Consistently, thermographic images of the mice taken with an infrared thermal camera show strong heat maps of the tumors as compared to the animal body (Figure 4C). Hereby, the tumor temperatures were 43 °C and higher while the body temperatures only increased slightly reaching about 38 °C. Although the 24 h and Hya/24 h mice groups revealed temperatures secluded to the tumors predominantly, the mice in the 72 h group revealed a stronger dispersion of the heat from the tumor to the body of the mice (Figure 4C, 72 h). This suggests that the nanoparticles distribute to some organs of the mice when left for 72 h after injection before the first hyperthermia treatment. Interestingly, the heat dose (CEM43) of the tumors in minutes was highest in the 24 h group, followed by the 72 h group, whereas the lowest heating dose was recorded for the Hya/24 h group (Figure 4D).

### 3.5. Computer Tomographic Images Indicate Hyaluronidase-Induced Infiltration of Dex-MNP in Tumor Models in Mice

**Figure 5:**
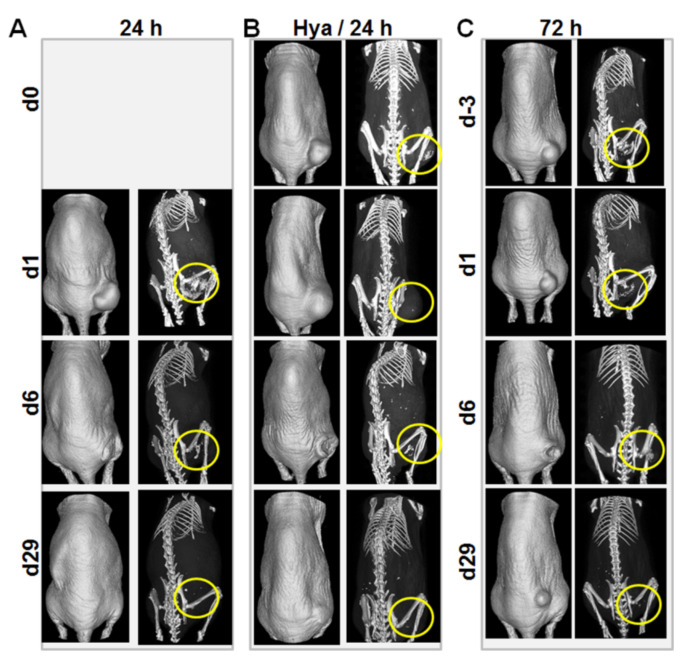
Representative micro-CT images of mice showing Dex-MNP deposits after intratumoral application and after hyperthermia treatment. All mice received comparable concentrations (0.5 mgFe/100 mm^3^ tumor volume) of intratumoral Dex-MNP, which reveal characteristic X-ray densities indicated with yellow circles. (**A**) Representative mouse of the 24 h group showing Dex-MNP at 24 h after application (d1) and at days 6 and 29 (d6/d29) after the first hyperthermia treatment. (**B**) Representative mouse of the hyaluronidase treatment group showing Dex-MNP immediately after (d0) and 24 h (d1) after injection, and at days 6 and 29 (d6/d29) after the first hyperthermia treatment. Hyaluronidase I-S (40 µg/100 mm^3^ tumor volume) was injected 2 h prior to Dex-MNP injection. (**C**) Representative mouse of the 72 h group showing Dex-MNP immediately after (d-3) and 72 h (d1) after injection, and at days 6 and 29 (d6/d29) after the first hyperthermia treatment.

The heating pattern monitored in the mice and also the heat doses estimated, correlated well with the distribution of the Dex-MNP in tumors before the first hyperthermia treatment. This was validated by computer tomographic imaging of the mice immediately after Dex-MNP application and a few hours before the first hyperthermia treatment. Accordingly, mice that received the Dex-MNP alone for 24 h revealed the Dex-MNP signals secluded to the areas of intratumoral application 24 h after (Figure 5A). In contrast, the Dex-MNP in the hyaluronidase-injected mice infiltrated the tumor tissues hence giving a weak X-ray density (Figure 5B), whereas the Dex-MNP in the 72 h group was seen to leak into the interstitial space underneath the tumors and also under the skin of the mice further away from the tumors (Figure 5C). This localization of the Dex-MNP within the tumors is more evident in the transaxial tomographic sections of the mice as shown in the supplementary materials (Supplementary data S3). Considering these differences in the Dex-MNP distribution, and the heating patterns observed, it was interesting to know what overall effects the treatment modalities would have on the tumor growth regression over time after treatment. 

### 3.6. Combining Hyaluronidase and Magnetic Hyperthermia Cause a Gradual and Persistent Reduction in Pancreatic Tumor Models in Mice

**Figure 6:**
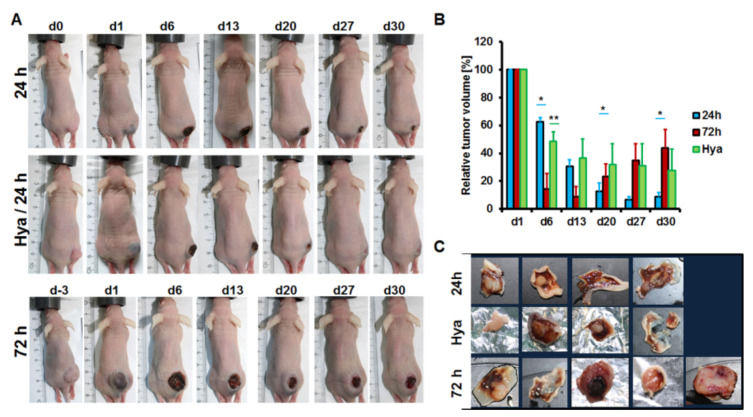
Representative images showing tumor growth inhibition after magnetic hyperthermia treatment alone or combined with hyaluronidase pretreatment. (**A**) Representative pictures of mice showing reduction in tumor volumes over time. (**B**) Relative tumor volumes of the different treatment groups over time. Each bar represents the mean of tumor volumes as percentage of the starting tumor volumes. n = 4/standard error of the mean (SEM) for the 24 h and Hya/24 h groups and n = 5/SEM for the 72 h group. * p < 0.05 for tumor growth differences between 24 and 72 h groups at day 6, 20, and 30. ** *p* < 0.04 for tumor growth differences between Hya/24 h versus 72 h group at day 6 and for Hya/24 h versus 24 h group at day 27. (**C**) Photographs of residual tumors excised on the final day (d30) of treatment monitoring substantiate the size of remaining tumor volumes determined by caliper measurements.

We further monitored the tumor growth in mice weekly for a period of 30 days from the day of the first hyperthermia treatment. Tumor growth inhibition was evident in all treatment groups after hyperthermia treatment (Figure 6A). This was most peculiar within the first 2 weeks of treatment and was significantly stronger in the 72 h group (*p* < 0.04) than the 24 h and Hya/24 h groups at day 6 and 13 after treatment (Figure 6B). However, whereas the 24 h and Hya/24 h groups experienced gradual and continuous decreases in tumor volumes over time till 30 days, the 72 h group revealed sudden regrowth of the tumors as from day 20 after hyperthermia (Figure 6B, red bars). Interestingly, the Hya/24 h group revealed a much slower tumor growth decrease over time than the 24 h group. This was, however, continuous till 30 days (Figure 6B, green bars) suggesting that a longer observation time would lead to a promising regression of the tumors. Furthermore, although the Hya/24 h group revealed no tumor regrowth at any time point, the 24 h group showed a slight tendency of tumor regrowth at day 30 as compared to day 27 (Figure 6B, blue bars), which was also evident in the size of the excised tumors at day 30 after treatment (Figure 6C).

### 3.7. Ultrathin Transmission Electron Microscopy of Tumor Tissues Reveals Enzyme-Modulated Stroma and Organelles Containing Dex-MNP

**Figure 7:**
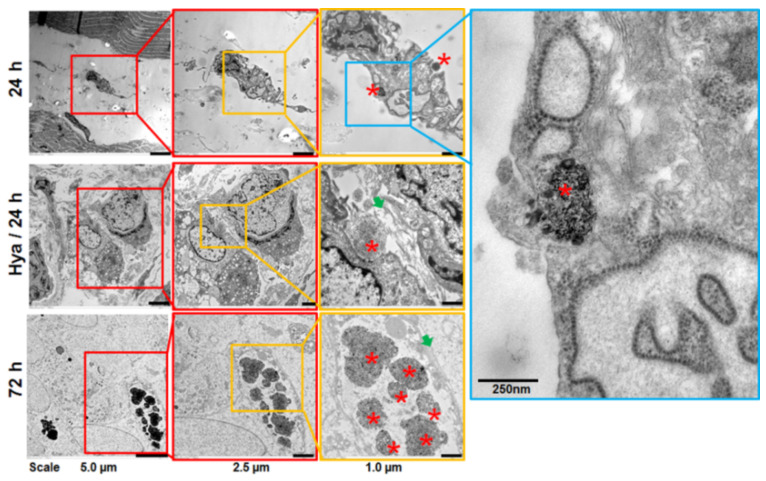
Ultrathin transmission electron micrographs of tumor tissues. The tumor tissue from the 24 h mice group show damaged tumor stroma and many invasive cells infiltrating the muscle. Likewise, tumor tissue from the hyaluronidase-treated Hya/24 h group show many intact tumor cells but a degraded and relaxed stroma with fragmented collagen fibers (green arrows). In contrast, the regrown tumor tissue from the 72 h mice group shows compactly arranged cells and stroma (green arrow). Some of the tumor and stromal cells in all groups show vesicles containing partly intact Dex-MNP (asterisks), which is especially high in the 72 h group.

Analyses of the residual and regrown tumor tissues by ultrathin transmission electron microscopy could pinpoint the localization of partly intact Dex-MNP in microvesicles within the tumor and stromal cells (Figure 7). Interestingly, the stroma of the 24 h and the hyaluronidase treatment group was strongly damaged by hyperthermia on one hand and by hyaluronidase on the other (Figure 7, green arrows). Contrarily, tumor tissue from the 72 h treatment group shows an intact stroma with compact cell–cell attachment in the regrown tumors (Figure 7, blue arrow). In all the tumor tissues analyzed, some tumor and stromal cells are seen with microvesicles of different sizes, which carry partially intact Dex-MNP (Figure 7, asterisks).

### 3.8. Effect of Intratumoral Hyaluronidase, Dex-MNP, and Magnetic Hyperthermia of Subcutaneous Tumor Models on the Blood Components of Mice

**Figure 8:**
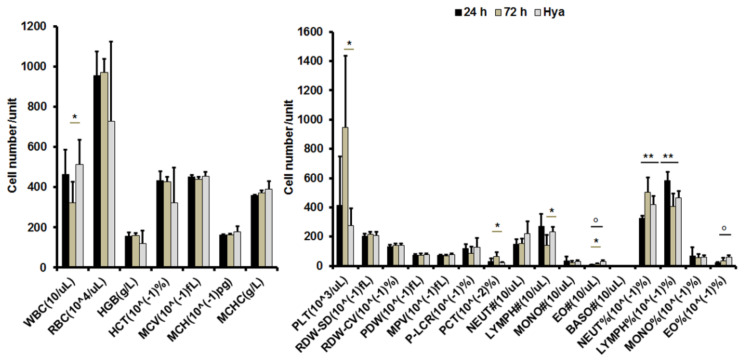
Comparative analysis of blood components of mice at day 30 after magnetic hyperthermia treatment without or with pretreatment with hyaluronidase. Each bar depicts the mean of blood components collected from n = 4 mice for the 24 h and Hya/24 h groups and n = 5 for the 72 h group and the respective standard deviations. * *p* < *0.05* for decreased white blood cells (WBC) and lymphocytes concentration (LYMPH#) in 72 h compared to the 24 h and Hya/24 h group and also increased platelets (PLT), platelet volume (PCT), and eosinophil concentration (EO#) in the 72 h versus Hya/24 h group. ** *p* ≤ 0.02 for lower percent of neutrophils (NEUT%) and higher percentage of lymphocytes (LYMPH%) in the 24 h compared to the Hya/24 h and 72 h groups. *° p* < 0.01 for higher concentration and percentage of eosinophils (EO# and EO%) in the Hya/24 h compared to the 24 h group.

One key emphasis of the underlying study was to verify whether the presence of intratumorally applied hyaluronidase and Dex-MNP would influence the overall levels of the blood components of mice after hyperthermia treatment. In this regard, capillary blood was collected from mice on day 30 after treatment and analyzed on a blood automation system as stated in the methods section. Interestingly, the white blood cells and lymphocyte concentration in the 72 h mice group were significantly (* *p* < 0.05) lower than in the 24 h and Hya/24 h groups, whereas the platelets, platelet volume, eosinophils, and percentage of neutrophils were significantly higher as compared to the 24 h and the Hya/24 h groups (Figure 8). Furthermore, the neutrophils and eosinophils concentration (EO#) and percentage (EO%) were significantly (° *p* < 0.05) higher and the percentage of lymphocytes was lower in the Hya/24 h mice group than the 24 h group (Figure 8).

## 4. Discussion

Besides the magnetic field strength (alternating current (AC) frequency and amplitude) of systems used for magnetic hyperthermia, the heating potential (specific absorption rate, and intrinsic loss power) and amount of magnetic materials deposited within tumors and tumor cells greatly influence the efficiency of magnetic hyperthermia treatment of the cells and tumors. The hydrodynamic diameter and charge (determined by the surface coating) influence the level of nanoparticles that are taken up by tumor cells, whereas tumor cells themselves convey heterogenic properties that can make them resistant to the uptake of magnetic particles. Furthermore, for intratumorally injected magnetic nanoparticles, it is vital to ensure a homogeneous infiltration and distribution in order to obviate heat spots and tumor areas with ineffective heating, which can result in tumor regrowth. This is especially vital for desmoplastic tumor types with highly resistant tumor and tumor stromal cells, e.g., the pancreatic ductal adenocarcinoma. This study focused on strategies that modulate magnetic nanoparticle infiltration in tumors and tumor cells in order to provide their efficient depletion and a relapse-free magnetic hyperthermia treatment in the future. 

Two different magnetic nanoparticles were used for in vitro and the in vivo studies based on different characteristic features. Starch-coated iron oxide nanoparticle (FluidMAG/C11-D) termed MNP for simplicity, revealed Z-averages of 100 nm and core sizes of 15–20 nm, whereas dextran-coated multicore iron oxide nanoparticles (termed Dex-MNP) exhibited Z-averages of 149 and 50–75 nm average core sizes. The heating potential (intrinsic loss power, ILP) of the starch-coated MNP was 2.7 nHm^2^/kg, whereas that of Dex-MNP was 5.1 nHm^2^/kg. Both ILP values lie in the range of suitably heating magnetic fluids. The differences seen between the formulations are related to the particle size [35] and also their shapes, as demonstrated by others with quasi-spherical- and deformed cube (octopod)-shaped nanoparticles [36,37]. It could be demonstrated by nanoparticle immobilization studies using 1% agarose or 10% polyvinyl alcohol (PVA) that the ILPs of nanofluids decrease with degree of immobilization, hence a drop from free fluid towards 10% PVA [38]. Thus, in vitro studies were done with the starch-coated MNP, whereas the in vivo studies were carried out with the Dex-MNP, one additional reason being that the Dex-MNP due to a large hydrodynamic diameter could not be taken up by cultured cancer cells or macrophages in vitro.

### 4.1. Matrix-Modulating Enzymes Enhance Cellular Uptake of Magnetic Nanoparticles and Improve Magnetic Heating and Depletion of Cells In Vitro

In the in vitro validations, the effect of hyaluronidases and collagenase on the uptake of MNP was evident in an increase in the level of iron within the cells detected by Prussian blue staining of iron and also partly by atomic absorption spectroscopy quantification of whole iron. The ability of pancreatic cancer cells to take up more MNPs after treatment with the enzymes can be partly ascribed to their proteolytic effect on hyaluronic acid and collagen, which are secreted by the cells to make cell–cell contacts and the extracellular matrix.

Pancreatic cancer cells, e.g., the Panc-1 cell line are known to express a variety of collagens, including collagen I, [39], IV, V, VI, IX, XIII, XIV, XV, XXV, and XXVIII [32], which serve, in general, as extracellular or transmembrane structural proteins in forming connective tissues, controlling angiogenesis, tumor invasion, and progression [40,41]. The increased expression of ECM components in Panc-1-derived 3D spheroids could be seen in the stiffness of the spheroids. Thus, the degradation of collagens in the cultured Panc-1 cells or their 3D spheroids by collagenase loosens the cell–cell attachments and inhibits the influence of the collagens on the penetration of MNPs or their uptake by the cells. This is evident in the disaggregated state of the monolayer cells or cells in 3D spheroids treated with collagenase and also the localization of the MNP in cells lying deep in the spheroids (see Figure 2). 

It is also conceivable that the hyaluronidase cleavage of hyaluronic acid (HA), which is also known to be endogenously expressed by many pancreatic cancer cell lines including the Panc-1 cells and 3D spheroids thereof [39] exerts effects on the cell–cell contacts and extracellular matrix amongst others, though at a lower degree as compared to collagenase in 3D spheroids. Hyaluronidases degrade HA in the extracellular matrix by proteolytic cleavage of the glycosidic bond between D-glucoronic acid and N-acetyl-D-glucosamine. Consequently, the interstitial pressure in the ECM decreases [42]. This decrease in the interstitial pressure was shown to be indirectly proportional to the hydraulic conductivity or permeability of drugs in the ECM, (the lower the pressure, the more/better drugs can permeate cells/tissues) [43]. Furthermore, the hydraulic permeability is indirectly proportional to the level of glycosaminoglycan present on the tumor cells, and are elevated in the tumor ECM [44]. A high endogenous expression of HA in Panc-1-derived 3D spheroids is known [39]. The fact that Panc-1-derived 3D spheroids revealed a less disaggregated cell–cell contact after treatment with hyaluronidase as seen with collagenase further substantiates the suitability of hyaluronidases for the gentle loosening of the stroma as opposed to collagenase. The uptake of MNPs by cells lying close to the surface of the 3D spheroids where immediate access to the enzyme and MNP was possible, and also by cells lying at the core of the 3D spheroids exposed the effect of hyaluronidases on loosening of the ECM and enhancing MNP infiltration. In comparison, MNP was seen only in the cells lying on the surface of 3D spheroids treated with MNP without enzymes.

The hyaluronidase- and collagenase-improved uptake of MNP by Panc-1 cells was further detected indirectly by the speed at which the cells attained 43 °C when exposed to an alternating magnetic field at the same AC frequency and amplitude. Enzyme and MNP-treated cells attained and exceeded 43 °C within less than 2 min and required a decrease in the amplitude in order to maintain approximately 43 °C for the overall treatment duration of 60 min. This indicates the presence of a high concentration of the MNP within the cells and supports the fact that the concentrations of magnetic nanomaterials within cells can be improved using matrix-modulating enzymes. Furthermore, the increased efficiency of magnetic heating in the cells treated with either hyaluronidase or collagenase and MNP before hyperthermia induced a higher cell death as compared to cells exposed to MNP alone and hyperthermia (see Figure 3). Thereby, the use of hyaluronidase I-S showed a more significant effect on hyperthermia-based cell depletion as compared to the hyaluronidase IV-S and collagenase. The fact that hyaluronidase I-S based influence on heat dose was lower as compared to hyaluronidase IV-S, but caused higher cell death and lower colony survival than the later, suggests that hyaluronidase I-S itself probably exerts longer lasting effects on the cells than hyaluronidase IV-S. Although further validation of this observation was out of the scope of this study, it is imaginable that hyaluronidase I-S cleavage of HA in human Panc-1 cells play similar signaling roles like the human hyaluronidase-I (HYAL1). Humans express five functional hyaluronidases (HYAL1-HYAL5) and a sixth nonfunctional pseudogene [45,46], which are responsible in regulating the level of HA expression and metabolism [47]. Amongst these human proteins, HYAL1 and HYAL2 have the most prominent expressions in tissues and are responsible for regulating HA turnover in cells and the ECM [48]. HYAL2 presents as a glycosylphosphatidylinositol- (GPI-) anchored protein and cleaves high-molecular weight HA (that is predominantly bound to CD44) within the ECM into variable lengths of smaller molecular weight polysaccharides [49]. These are internalized and further hydrolyzed by HYAL1 to smaller molecules [47], causing reduction in HA in the ECM, reduced interstitial pressure and enhanced permeability of the ECM as a consequence. Reports substantiate high expressions of endogenous HYAL2 and HYAL3 in different pancreatic cancer cell lines including the Panc-1, and a negligibly low level of HYAL1 [31]. Hence, it is likely that the use of hyaluronidase I-S as implemented herein contributes in completing the endogenous HYAL2 initiated HA turnover regulation, leading to cell death, which otherwise is repressed by the malignant PDAC phenotype. This is probably because the influx of HYAL2-hydrolyzed HA into cells is repressed if further degradation and clearance within the cells is not possible due to the absence of HYAL1, e.g., [48].

Interestingly, cells treated with collagenase alone revealed a slight proliferative effect of the enzyme on the cells, which, in part, correlates with reports demonstrating the proliferative effect of collagenases on keratinocytes [50] during wound healing. This could, in part, contribute in counteracting the hyperthermia effect resulting from improved MNP uptake and hyperthermia treatment seen in this study.

### 4.2. Hyaluronidase Enhances Infiltration of Magnetic Nanoparticles into the Stroma of Panc-1 Tumors, and Their Slow and Effective Treatment by Magnetic Hyperthermia 

In the in vivo situation, other components contribute in making the TME and ECM more complex. Hence, the preliminary goal was to validate whether the use of hyaluronidase would contribute in relaxing the tumor stroma, improve the infiltration of magnetic nanoparticles and a subsequent magnetic hyperthermia treatment, and tumor depletion. The large dextran-coated Dex-MNP, which could not be taken up by cultured cancer cells and macrophages were used in order to suitably monitor their deep infiltration and distribution in the tumors. Consistently, mice that received intratumoral hyaluronidase 2 h before the injection of Dex-MNP revealed weaker and dispersed Dex-MNP densities on CT images that were indicative of their infiltration and distribution within the tumors 24 h after injection (see Figure 5). Furthermore, the overall heat dose (CEM43° of the tumor surfaces) determined for the hyaluronidase-treated tumors was lower than that achieved with the injection of Dex-MNP alone. This was opposed to observations acquired with cultured cells, and sounds intriguing at a first glance. However, considering that the tumor temperatures are measured from the surface, and that the infiltration and distribution of the Dex-MNP within the tumors means reduction in the Dex-MNP deposit per unit area, it is expected that the measured temperatures of the tumors should be lower and more homogenously distributed within the tumor with increase in infiltration. Thus, the reduced heating further substantiates ECM loosening and reduction in the interstitial pressure [42,43,44], due to hyaluronidase I-S action, and the improved infiltration of Dex-MNP into the tumor stroma as a consequence.

Interestingly, waiting 72 h after the injection of Dex-MNP before conducting the first magnetic field exposure also revealed differences in the distribution within the tumors. The Dex-MNP were seen to have leaked out of the injection positions and settled underneath and around the tumors at 72 h after injection without pretreatment with hyaluronidase (see Figure 5 and Supplementary data S3), rather than distribute within the tumors as was the case after hyaluronidase application. Hence, the actual tumor temperatures achieved underneath the tumors in the 72 h group was probably higher than recorded from the tumor surfaces. This is supported by the rapid disappearance of the tumors within the first 2 weeks of therapy (See Figure 6) and also the effect of the therapy on the systemic increase in platelet number and volume, as well as eosinophils and neutrophils in the 72 h group as opposed to the 24 h or Hya/24 h groups. These blood components are involved in blood coagulation, injury-related inflammation, and tissue repair [51,52] and hence likely contribute in tumor regrowth. The fact that regrowth of the tumors in the 72 h group were evident from day 20 onwards suggests that rapid and ablative destruction of the tumors and underlying blood vessels is disadvantageous for relapse-free tumor depletion. This is because resistant tumor cell niches may acquire a more aggressive phenotype leading to the regrowth observed. Thus, the distribution of the Dex-MNP in the 72 h group and the overall effect on hyperthermia and tumor depletion further supports the role of the implemented hyaluronidase I-S on the reduction in HA within the tumor stroma of the Hya/24 h mice group, and the reduction in the tumor interstitial pressure [42]. The reduced interstitial pressure, in turn, enhances permeability of the tumor stroma [43,44] to Dex-MNP. Despite the increased infiltration and consequent low overall heat doses acquired with the use of hyaluronidase, it was hypothesized that tumor growth inhibition would take place and tumor regrowth, which has been repeatedly observed when conducting mild hyperthermia alone [34,53], would also be circumvented. Consistently, tumor depletion was slow but persistent, due to the effects of hyaluronidase I-S. In effect the tumor volumes as from day 13 of treatment onwards were comparably bigger for the Hya/24 h group than the 24 h group, yet reduced continuously till day 30 of treatment. In contrast, the 24 h mice showed slight increases in the tumor volume between day 27 and day 30 of treatment, suggesting the beginning of tumor regrowth in some mice. This was also supported by electron micrographs of the residual tumors of the 24 h group, which detected invasive cells containing Dex-MNP infiltrating the underlying muscle (see Figure 7, 24 h). Taken together, these results suggest that a homogenous distribution of magnetic materials and hence heat within tumors results from the degradation of HA by hyaluronidase and a consequent relaxation of the tumor stroma, and represents a beneficial strategy for a slow and relapse-free hyperthermia treatment. This is probably cancer type dependent, since the endogenous hyaluronidase expression varies in different tumor cells [31,48]. Hence, the choice of hyaluronidase to be implemented must be selected based on the malignant phenotype and their HYAL requirement. In the underlying work, the hyaluronidase I-S compensated the negligible level of HYAL1 in Panc-1 cells [31] and triggered continuous breakdown of endogenous HYAL2-hydrolyzed HA fragments, thereby contributing to a reduced HA level in the stroma. Conjugation of hyaluronidase I-S to a fluorescent dye showed their internalization into Panc-1 cells (not shown), which substantiates the observations made in the underlying studies. Moreover, ultrathin sections of residual tumors analyzed by electron microscopy validated a highly disaggregated stroma of tumors from the Hya/24 h group as opposed to the stroma of regrown tumors from the 72 h group.

Besides the disaggregation of cell–cell contacts and relaxation of the tumor stroma, the injected hyaluronidase is expected to inhibit the HA-induced immunosuppressive reaction of the tumor microenvironment, by keeping an overall low HA level in the ECM. It has been shown in different tumors, including pancreatic cancers, that HA in the tumor stroma interacts with a variety of receptors including CD44 and triggers the activation of cancer stem cell markers such as OCT4, Nanog, and Stat-3, as well as a diverse range of growth factors and cytokines, which promote cancer stem cells renewal and expansion, chemoresistance, and an enhanced tumor relapse as a consequence [6,7,13,31,54]. Thus, it is conceivable that combining the effects of hyaluronidase with hyperthermia to achieve the gradual and persistent tumor growth inhibition observed here represents a suitable strategy to achieve a relapse-free therapy of pancreatic cancers in the future. Furthermore, the use of hyaluronidase has enhancing effects on the efficacy of many therapeutic drugs [13,54,55], including gemcitabine and paclitaxel [56], which are known therapeutics used for pancreatic cancers and can be further combined with hyperthermia. 

## 5. Conclusions

The underlying study demonstrates that hyaluronidases and collagenase enhance the uptake of magnetic nanoparticles by the chemoresistant Panc-1 cells in culture and thereby increase the concentration required for a suitable magnetic hyperthermia and cell depletion. The use of hyaluronidase I-S in in vivo mice studies enabled tumor ECM loosening and a consequent intratumoral infiltration and distribution of Dex-MNP, which due to a large hydrodynamic diameter of 149 nm could not be taken up by cultured cancer cells or macrophages in vitro. The consequence of the infiltration and improved distribution of Dex-MNP within the tumors was a controlled distribution of heat leading to comparably lower heat doses, yet a gradual and persistent tumor growth inhibition over time and the circumvention of tumor regrowth. This was based on the compensation of the repressed HYAL1 expression in Panc-1 cells, by hyaluronidase I-S, which cleaves and reduces stromal HA levels and interferes with their receptor interactions and signaling. Thus, we are convinced that combining the hyaluronidase-enhanced magnetic hyperthermia as adjuvant therapy with other therapies will be a promising strategy to eradicate many solid cancers including pancreatic cancers in the future. 

## Data Availability

The data reported in this study is available upon request from the corresponding authors.

## Supplementary Materials

The following are available online at https://www.mdpi.com/2079-4991/11/2/438/s1, Supplementary data S1: Concentration dependency of uptake of starch-coated MNP (FluidMAG/C11-D) in Panc-1 cells, Supplementary data S2: A flake-shaped dextran-coated iron oxide nanoparticle with a hydrodynamic diameter of 149 nm cannot be taken up by macrophages and pancreatic cancer cells in vitro; Supplementary data S3: mCT transaxial images show intratumoral distribution of large dextran-coated MNP deposits.
