# Supplementary material for: Effect of Matrix-Modulating Enzymes on the Cellular Uptake of Magnetic Nanoparticles and on Magnetic Hyperthermia Treatment of Pancreatic Cancer Models In Vivo
Source: Nanomaterials (Basel). 2021 Feb 9;11(2):438. doi: 10.3390/nano11020438 (PMC7915425; doi:10.3390/nano11020438)
Supplement: Supplementary file 1 [file nanomaterials-11-00438-s001.pdf]

nanomaterials

Supplementary material

Effect of Matrix-Modulating Enzymes on The Cellular Uptake of Magnetic Nanoparticles and on Magnetic Hyperthermia Treatment of Pancreatic Cancer Models In Vivo

Felista L. Tansi [1,*], Filipp Fröbel [1], Wisdom O. Maduabuchi [1], Frank Steiniger [2], Martin Westermann [2], Rainer Quaas [3], Ulf K. Teichgräber [4] and Ingrid Hilger [1,*]

[1] Department of Experimental Radiology, Institute of Diagnostic and Interventional Radiology, Jena University Hospital - Friedrich Schiller University Jena, Am Klinikum 1, 07747 Jena, Germany; filipp.froebel@yahoo.de (F.F.); wisdom.maduabuchi@med.uni-jena.de (W.O.M.); ulf.teichgraeber@med.uni-jena.de (U.K.T)
[2] Center for Electron Microscopy, Jena University Hospital - Friedrich Schiller University Jena, Ziegelmuehlenweg 1, 07743 Jena, Germany; frank.steiniger@med.uni-jena.de (F.S.); martin.westermann@med.uni-jena.de (M.W.)
[3] chemicell GmbH, 12103 Berlin, Germany; quaas@chemicell.de
[4] Institute of Diagnostic and Interventional Radiology, Jena University Hospital - Friedrich Schiller University Jena, Am Klinikum 1, 07747 Jena, Germany; ulf.teichgraeber@med.uni-jena.de
* Correspondence: felista.tansi@med.uni-jena.de (F.L.T.); Tel. +49-3641-9324993 (F.L.T.); ingrid.hilger@med.uni-jena.de (I.H.) Tel. +49-3641-9325921 (I.H.)

Supplementary data S1: Concentration-dependency of uptake of starch coated MNP (FluidMAG/C11-D) in Panc-1 cells

Panc-1 cells grown on 8-well chambered culture slides were supplemented with different concentrations of starch coated magnetic nanoparticles (FluidMAGC11-D) as described in the methods section of the article and cultured for 24 h at standard culture conditions. The cells were then harvested and the nanoparticulate iron stained by the Prussian blue staining method and images acquired on a light microscope (Olympus BX50) at a 40x magnification (Figure S1).

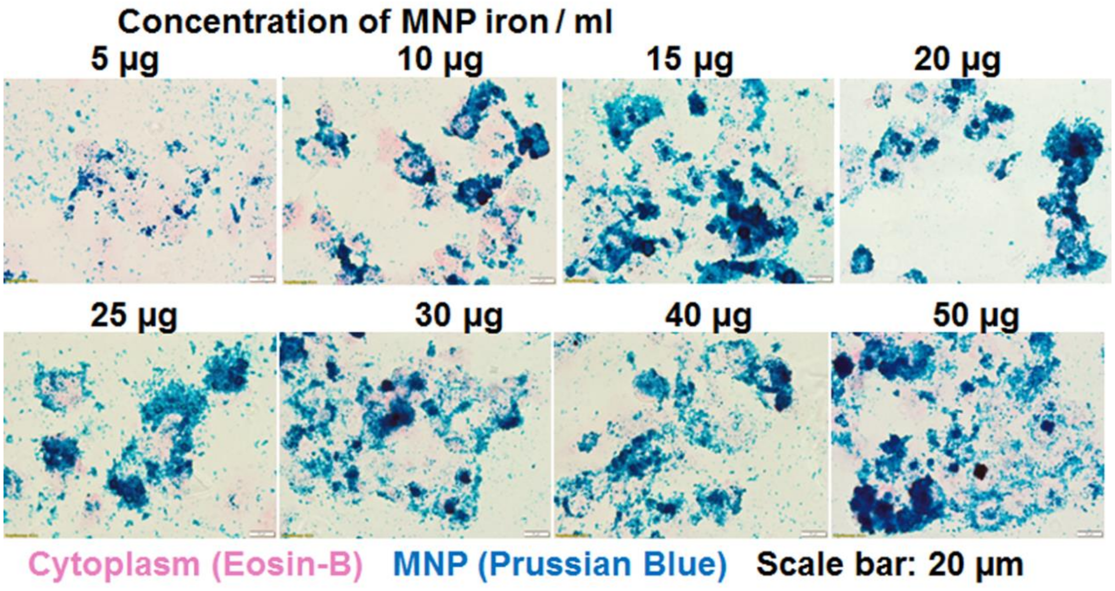

Figure S1: Light microscopic images of Panc-1 cells showing different levels of nanoparticle based iron after 24 h incubation with different MNP concentrations. The most reliable uptake of the MNP is seen when implementing a concentration of 10 µgFe / mL culture medium.

Supplementary data S2: A flake-shaped dextran coated iron oxide nanoparticle with a hydrodynamic diameter of 149 nm cannot be taken up by macrophages and pancreatic cancer cells in vitro.

Panc-1 cells or the murine macrophage cell line J774.1 were grown on 8-well chambered culture slides and exposed to the dextran coated iron oxide nanoparticles (RCL01, termed Dex-MNP) at a concentration of 50µg Fe /ml culture medium for 24 h at standard culture conditions. The cells were then harvested and the nanoparticulate iron stained by the Prussian blue staining method and images acquired on a light microscope (Olympus BX50) at a 40x magnification. As can be seen from the images, the cells revealed no stain of the iron indicating the inability of the cells to take up the flake-shaped large Dex-MNP (Figure S2).

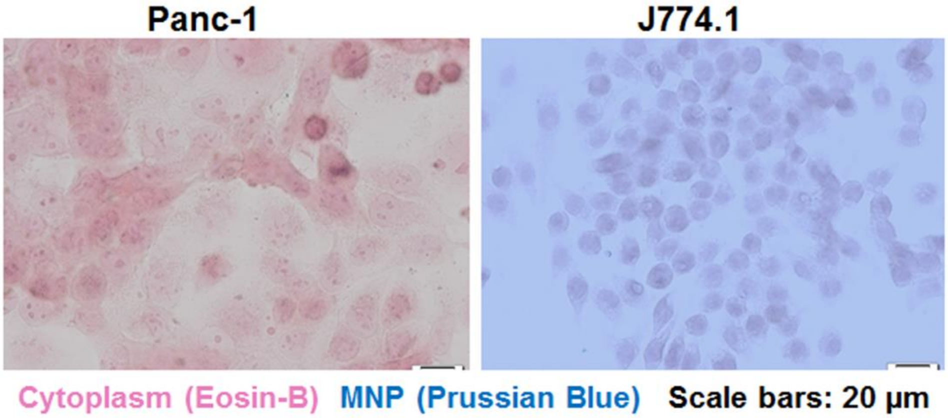

Figure S2: Light microscopic images of Panc-1 and murine macrophage J774.1 cells incubated for 24 h with Dex-MNP (50 µgFe / ml) and stained for iron by the Prussian blue method. The absence of the blue stain of iron in the cells is indicates the inability of the cells to take up the Dex-MNP.

Supplementary data S3: mCT Transaxial images show intratumoral distribution of large dextran-coated MNP deposits

Mice were injected intratumorally with the Dex-MNP either without (24 h and 72 h groups) or after 2 h pre-treatment with hyaluronidase 1-S and then imaged at different time points on the IVIS-Spectrum CT in order to detect the Dex-MNP which have higher X-ray densities than the mice and tumor tissues. As seen from the transaxial images of the mice at the positions of the tumors, pre-treatment of the tumors with hyaluronidase enables infiltration and distribution of the Dex-MNP within the tumor, hence a faint and diffuse Dex-MNP density at 24 h after injection (Figure S3, Hya / 24 h, d1) as opposed to injection without hyaluronidase whereby the Dex-MNP density is seen predominantly at the position of injection with mild leakage underneath the tumors after 24 h (Figure S3, 24 h, d1). Waiting 72 h after injection of Dex-MNP alone leads to leakage of the Dex-MNP from the position of injection, into the space underneath and around the tumor (Figure S3, 72 h, d1).

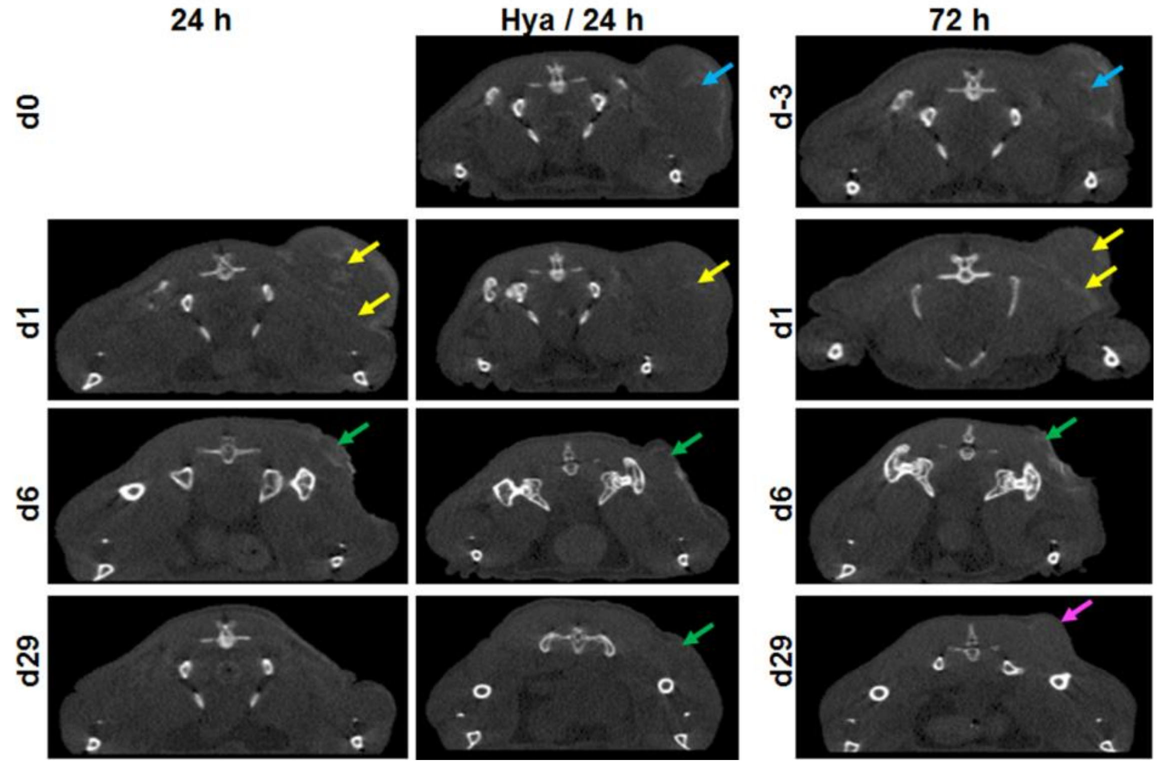

Figure S3: Computer tomographic transaxial images of mice flank showing the intratumoral distribution of flake-shaped dextran-coated Dex-MNP deposits. The blue arrows depict the white signals of the Dex-MNP on the day of injection (d0 for the 24 h and Hya / 24 h groups, or d-3 for the 72 h group). Yellow arrows point at the location of the Dex-MNP at 24 h after injection (for the 24 h and Hya / 24 h groups) or 72 h after application (for the 72 h group). Green arrows depict residual tumors at day 6 (d6 or d29) after the first hyperthermia treatment and lilac arrow points at a regrown tumor in the 72 h group at day 29 after the first hyperthermia. Hyperthermia exposures took place on day 1 (d1) after CT imaging and on day 7.
